# Protective Effect of Compound Formula Rehmannia against Neurotoxicity and Apoptosis Induced by *α*-Syn in *In Vivo* and *In Vitro* Models of Parkinson's Disease

**DOI:** 10.1155/2020/5201912

**Published:** 2020-08-14

**Authors:** Long Teng, Minchun Yang, Xiaoqing Jin, Lu Qian, Weijia Yang, Xuanxuan Ren, Qing Qu

**Affiliations:** ^1^Department of Internal Medicine of Traditional Chinese Medicine, Zhejiang Hospital, Hangzhou, Zhejiang 310030, China; ^2^Department of Massage, Zhejiang Hospital, Hangzhou, Zhejiang 310030, China

## Abstract

The present study aimed to investigate the protective effect of compound formula Rehmannia (CFR) against the development of Parkinson's disease (PD). After the *in vivo* and *in vitro* models of PD were established with overexpression *α*-syn induced, CFR was administrated into the PD model rats for 6 weeks or SK-N-SH cells with coincubation for 48 h. Apomorphine-induced rotation test, CCK8 assay, TUNEL assay, immunofluorescence staining, and western blot assay were performed to evaluate the behavioral changes, cell viability, cell apoptosis, *α*-syn, GSK-3*β*, P-GSK-3*β* (Ser9), P-GSK-3*β* (Tyr216), and *β*-catenin expression in PD rats or SK-N-SH cells. PD rat behavior results showed that the rotation numbers were significantly decreased in the CFR treatment group comparing with the AAV-*α*-syn PD model group. The cell viability suppressed by H_2_O_2_ and *α*-syn in SK-N-SH model cells was also significantly improved with CFR administration. Cell apoptosis and *α*-syn overexpression observed in PD rats and SK-N-SH cells were also inhibited by CFR treatment. Furthermore, the protein expression of *α*-syn, GSK-3*β*, P-GSK-3*β* (Ser9), P-GSK-3*β* (Tyr216), and *β*-catenin in *in vivo* and *in vitro* was also significantly regulated by CFR. The present study suggested that CFR may be considered as a potential neuroprotective agent against PD, and this application will require further investigation.

## 1. Introduction

Parkinson's disease (PD) is a common neurodegenerative disease prevalent in middle-aged and elderly populations. Clinical features of PD are a range of motor and nonmotor symptoms, such as static tremor, bradykinesia, rigidity, postural balance disorder, depression, abnormal sleep behavior, and memory loss [1]. The epidemiological studies report that the prevalence of PD is about 0.3%, while the incidence is doubled with the incidence of 1% to 2% in the elders over 65 years old and 3% to 5% in the elders over 85 years old [2]. PD is characterized by the progressive degeneration of dopaminergic neurons in the substantia nigra pars compacta (SNpc) and loss of dopamine (DA) in the striatum [3]. Additionally, oxidative stress and mitochondrial dysfunction are also involved in the degeneration and loss of dopaminergic neurons in the substantia nigra. DA replacement therapy represented by levodopa (L-Dopa) remains the most applied method in PD treatment. However, still PD cannot be cured completely yet, and more importantly, the side effects of L-Dopa gradually appear with the prolongation of time. Talking of the side effects, the most seriuos one is the L-Dopa-induced dyskinesia, the clinical manifestation mainly resembles dance, which seriously affects the quality of daily life of PD patients and creates an escalating burden on the family and society [4].

The pathogenesis of PD is complex, and the first protein identified to be associated with PD is *α*-synuclein (*α*-syn), which is abundant in neuronal inclusions termed Lewy bodies and Lewy neurites. The mutation or overaccumulation of *α*-syn causes abnormal aggregation and misfolding, which damage the normal physiological functions of cells, promote cell death, inhibit the release of DA, and increase the likelihood of neurodegenerative changes [5]. Due to the close correlation between *α*-syn and PD, *α*-syn is considered as a potential therapeutic target for neurodegenerative diseases, especially for PD. And glycogen synthase kinase-3*β* (GSK-3*β*), one of the GSK-3 isomers, is directly associated with the neuronal apoptosis, and its inhibition also affects the expression of *α*-syn [6]. It stimulates cell survival transcription factors such as CRE-binding protein (CREB), nuclear factor of the activated T-cell (NFAT) protein family, and *β*-catenin. Study on differentiated SH-SY5Y cells in oxygen and glucose deprivation (OGD) model also found that GSK-3*β* inhibitors had antiapoptotic activity, as evidenced by reduced caspase-3 enzyme activity and increased survivin transcription, and GSK-3*β* also upregulated survival AKT1/GSK-3*β*/*β*-catenin pathway, GAP43, Ngn1, and NeuroD2 gene transcription to maintain neuronal survival [7]. However, the effect of *α*-syn overexpression on GSK-3*β* activity and the role of the GSK-3*β*/*β*-catenin signaling pathway in neurodegenerative diseases are still unclear.

Traditional Chinese medicine (TCM) combined with Western medicine has advantages of “effect-enhancing and toxicity-reducing” in the treatment of PD [8]. TCM can attenuate the progression of the disease and the occurrence of the complications to a certain extent, which play a therapeutic role for PD patients. The compound formula Rehmannia (CFR) is a formula based on the holistic view and syndrome differentiation theory of TCM; the protective effect against PD is also proved through long-term clinical and experimental studies [9]. In this study, based on the previous research, we suspected that neuroprotective effect of CFR against PD might be correlated with *α*-syn and GSK-3*β*/*β*-catenin signaling, *in vivo* and *in vitro* models of PD induced with *α*-syn were established, and neuroprotective effect and possible mechanism of CFR were also explored.

## 2. Materials and Methods

### 2.1. CFR Decoction and CFR-Contained Serum Preparation

CFR, composed by prepared *Rehmannia glutinosa* 15 g, *Concha Margaritifera Usta* 30 g, fried *Salvia miltiorrhiza* 9 g, *Acorus gramineus Soland* 12 g, and green tea 6 g, was boiled to obtain the CFR decoction with a concentration of 5.18 g/ml for further *in vivo* experiment. The CFR decoction preparation was processed by Hangzhou Huadong Medicinal Materials Co., Ltd. (Zhejiang, China). SD rats weighing 250–300 g were intragastric administrated with CFR at a dose of 9 ml/kg/d. After treatment for 3 days, blood was obtained at 1 h after the last administration and centrifuged at 2,000 r·min^−1^ for 5 min at room temperature. The upper serum fraction was isolated and further inactivated at 56°C for 30 min. The serum was then filtered through a 0.22 *μ*m filter, and these obtained CFR-contained serum samples were stored at −70°C for further *in vitro* experiment.

### 2.2. Animals

Male SD rats, weighing 250–300 g, were purchased from Shanghai Sipper-BK Experimental Animals Co., Ltd. (Shanghai, China; certification no. SCXK: 2013-0016). All rats were housed in cages under controlled condition of temperature (22 ± 2°C), humidity (50%–60%), and 12 h light/dark cycle lighting with food and water freedom in Zhejiang Traditional Chinese Medicine University, Animal Experimental Research Center (Zhejiang, China; certification no. SYXK: 2013-0184). All animal procedures were approved by the animal ethics committee of Zhejiang Traditional Chinese Medicine University.

### 2.3. Surgical Procedure and Experiment Design

SD rats were randomly assigned into five groups with 10 rats in each group. Control group: rats were fixed to a stereotaxic frame without any surgical procedures; sham-operated group: rats were injected with 0.2% vitamin c; negative control group: rats were injected with AAV empty vector and intragastric injected with normal saline for 6 weeks; PD model group: rats were injected with AAV-*α*-syn and intragastric injected with normal saline for 6 weeks; and PD + CFR group: rats were injected with AAV-*α*-syn and intragastric administrated with CFR at 9 ml/kg with 2 times/d for 6 weeks. PD model surgical procedures were performed according to the previous described study with slight modifications [10]. AAV-*α*-syn and AAV empty vector were purchased from Genechem (China). After the anesthesia with intraperitoneal injection of 3% pentobarbital sodium 40 mg/kg, SD rats were fixed to a stereotaxic frame, and then 10 *μ*l of AAV-*α*-syn, AAV empty vector, or 0.2% vitamin c were injected to the substantia nigra. The injections were carried out to the substantia nigra at the following coordinates: anterior-posterior: 5.2 mm, medial-lateral: 1.0 mm, dorsal-ventral: 9.0 mm below the dural surface; anterior-posterior: 5.2 mm, medial-lateral: 2.5 mm, and dorsal-ventral: 8.5 mm below the dural surface [11]. Vector solutions were infused at a rate of 0.2 ml/min, and the needle was left in place for an additional 35 min before it was slowly retracted.

### 2.4. Apomorphine-Induced Rotation Test

Rotation test is the most commonly used method for assessing functional efficacy of the PD animal model. In this study, rotation test was performed according to a previous study [12]. In brief, the rats in different groups were injected with apomorphine (the dopamine agonist) 2 mg/kg, i.p., and the number of contralateral rotations (turn unilaterally 360 degrees) was counted for a period of 30 min.

### 2.5. Brain Tissue Preparation

At the end of the animal experiment, all rats were anaesthetized with 40 mg/kg 3% pentobarbital sodium and exsanguination. Subsequently, about 50 ml saline was perfused through the ascending aorta, and then brain tissues were dissected, fixed in 4% paraformaldehyde for 2 h and cryoprotected overnight in sucrose. Brain tissues were sectioned on a freezing microtome (Leica) at a thickness of 35 *μ*m carefully. Other brain tissue samples were stored at −80°C for further western blot experiment.

### 2.6. SK-N-SH Cell Culture and Treatment

SK-N-SH human neuroblastoma cells were purchased from iCell Bioscience, Inc. (Shanghai, China), cultured in DMEM containing 10% fetal bovine serum, 100,000 U/mL penicillin, and 100,000 *μ*g/L streptomycin, and maintained in a humidified incubator at 37°C with 5% CO_2_. For generation of the *α*-syn overexpression cell line, SK-N-SH cells were transfected with *α*-syn plasmids or empty control plasmids in six-well plates with Lipofectamine 2000 according to the manufacturer's protocol. Afterwards, cells were treated and incubated with 300 *μ*M H_2_O_2_ for 30 min to induce cell injury followed by treatment with low or high dose of CFR-contained serum (5%, 10%) for 48 h [13].

### 2.7. CCK8 Assay

CCK8 assay was used to evaluate the effect of the CFR on the cell viability against H_2_O_2_ and *α*-syn. SK-N-SH cells were plated into 96-well plates and incubated for 24 h 37°C with 5% CO_2_. After that, 5% or 10% CFR-contained serum was added to the SK-N-SH cells and coincubated for 24 and 48 h. Then, 10 *μ*l of CCK8 solution (Beyotime, China) was added to each well and further incubated for 4 h. Finally, the OD value was measured in a microplate reader (CMaxPlus, SpectraMax) at 450 nm.

### 2.8. TUNEL Assay

TUNEL assay was performed to assess the protective effect of CFR against *α*-syn-induced apoptosis in *in vivo* and *vitro* models of PD. In brief, TUNEL staining was performed using the TUNEL kit as instructed by the manufacturer (BBI, China). Brain tissue sections or SK-N-SH cells were transferred to the TUNEL reaction mixture for incubation; subsequently, the tissues or cells were subjected to 4′,6-diamidino-2-phenylindole (DAPI) for further staining. After that, all samples were visualized under an inverted microscope equipped with fluorescence (Zeiss, Germany), and three random fields were selected per sample.

### 2.9. Immunofluorescence Staining

To assess the expression of *α*-syn in SK-N-SH cells, immunofluorescence staining was conducted. SK-N-SH cells were fixed with 4% paraformaldehyde and washed with PBS 3 times, then permeabilized with 0.1% Triton X-100 for 15 min, and washed with PBS 3 times; 2% BSA was further added for 1 h of blocking and then washed with PBS 3 times. After that, cells were incubated with the primary anti-*α*-syn antibody overnight at 4°C, washed with PBS 3 times, and was followed by incubation with DAPI for 1 h at room temperature. Brain tissue images and cell images were obtained with an inverted microscope equipped with fluorescence.

### 2.10. Western Blot Assay

Rat brain tissues or SK-N-SH cells were collected and extracted with lysis buffer to obtain total protein. The protein samples were separated on 5% SDS-PAGE and then transferred to PVDF membranes. After blocked in 5% nonfat milk for 2 h at room temperature, the membranes were washed with TBST 3 times and then incubated with the primary antibodies against *α*-syn, GSK-3*β*, P-GSK-3*β* (Ser9), P-GSK-3*β* (Tyr216), and *β*-catenin (Abcam, USA) at 4°C overnight. After 3 times washing with TBST, the membranes were further incubated with secondary antibodies for 1 h at room temperature. The protein blots were visualized by using an ECL system and quantified with ImageJ software; GAPDH was used as a loading control to normalize the band density.

### 2.11. Statistical Analysis

All data were presented as mean ± standard deviation (SD). The collected data were subjected to one-way ANOVA followed by LSD test to analyse the statistical differences between groups. *P* < 0.05 was considered as statistically significant.

## 3. Results

### 3.1. Behavioral Assessment for the Effect of CFR on the PD Model Rat

**Figure 1:**
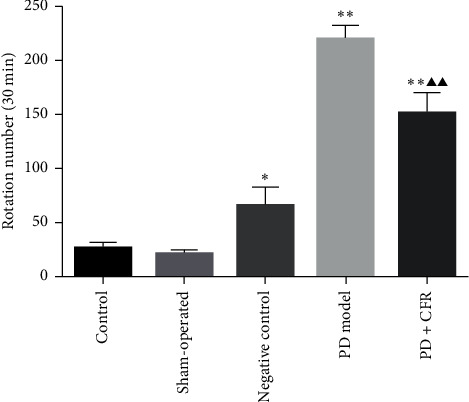
Effect of CFR on the rotation numbers of the rats (*n* = 10). Control group: without any surgical procedures or treatment, sham-operated group: 0.2% vitamin c, negative control group: AAV empty vector, PD model group: AAV-*α*-syn, and PD + CFR group: AAV-*α*-syn + CFR, 9 ml/kg, 2 times/d, 6 weeks. Compared with the control group, ^*∗*^*P* < 0.05 and ^*∗∗*^*P* < 0.01; compared with the PD model group, ^*▲*^*P* < 0.05 and ^*▲▲*^*P* < 0.01.

As observed, the control group showed an average of 25.7 ± 6.11/30 min rotation number, and the sham-operated group showed an average of 20 ± 2.82/30 min rotation number (Figure 1). However, in the PD model group, the average rotation number was 219 ± 13.52 in 30 min and was significantly increased compared with the control group (*P* < 0.01). Furthermore, with the administration of CFR for PD rats, the rotation number was 150 ± 20.1 in average and was decreased significantly compared with the PD model group (*P* < 0.01), but rotation numbers between PD + CFR and control groups were also statistically significant (*P* < 0.01).

### 3.2. Effect of CFR on the Apoptosis of the Substantia Nigra and Striatum Tissues of the PD Model Rat

**Figure 2:**
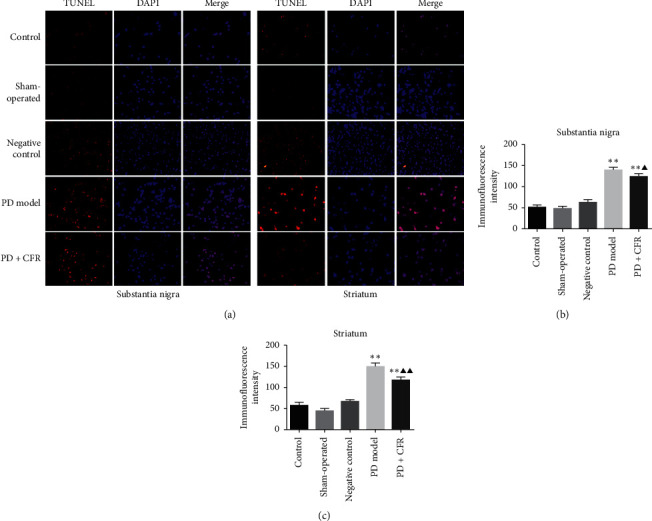
TUNEL assay for the effect of CFR on *α*-syn-induced apoptosis in the substantia nigra and striatum of the PD rat brain tissues (magnification, ×200). Control group: without any surgical procedures or treatment, sham-operated group: 0.2% vitamin c, negative control group: AAV empty vector, PD model group: AAV-*α*-syn, and PD + CFR group: AAV-*α*-syn + CFR, 9 ml/kg, 2 times/d, 6 weeks.

The antiapoptosis effect of CFR on the PD model rat was assessed using TUNEL assay. As shown in Figure 2, TUNEL-positive cells in the control group, sham-operated group, and negative group of the brain substantia nigra tissues could be observed with weak immunofluorescence intensity. TUNEL-positive cells in the PD model group were significantly increased compared with the control group, thus inducing the cellular apoptosis (*P* < 0.01). And CFR administration obviously reduced the cellular apoptosis with decreased amount of the TUNEL-positive cells compared with the PD model group (*P* < 0.05). In addition, similar tendency can also be observed for the antiapoptosis effect of CFR on PD model rat striatum tissues. TUNEL-positive cells in the PD model group were obviously increased compared with the control group, and the CFR treatment for the PD rat model decreased the TUNEL-positive cells compared with the model group (*P* < 0.01).

### 3.3. Effect of CFR on the Protein Expression of *α*-Syn, GSK-3*β*, P-GSK-3*β*, and *β*-Catenin in the PD Model Rat

**Figure 3:**
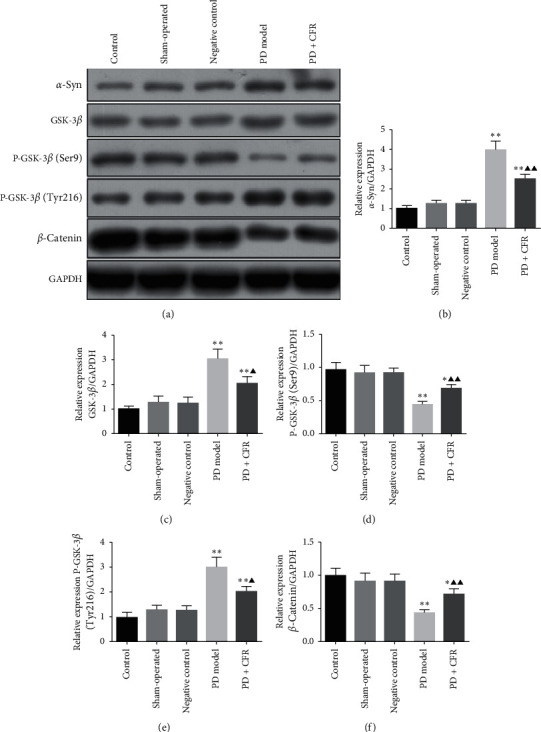
Effect of CFR on the protein expression of *α*-syn, GSK-3*β*, P-GSK-3*β* (Ser9), P-GSK-3*β* (Tyr216), and *β*-catenin in rat brain tissues. Control group: without any surgical procedures or treatment, sham-operated group: 0.2% vitamin c, negative control group: AAV empty vector, PD model group: AAV-*α*-syn, and PD + CFR group: AAV-*α*-syn + CFR, 9 ml/kg, 2 times/d, 6 weeks. Protein expression was determined using western blot, and GAPDH was used as a control. Compared with the control group, ^*∗*^*P* < 0.05 and ^*∗∗*^*P* < 0.01; compared with the PD model group, ^*▲*^*P* < 0.05 and ^*▲▲*^*P* < 0.01.

Western blot analysis was performed to determine the expression of *α*-syn, GSK-3*β*, P-GSK-3*β* (Ser9), P-GSK-3*β* (Tyr216), and *β*-catenin in experiment groups. As shown in Figure 3, there were no significant differences for the protein expression of *α*-syn, GSK-3*β*, P-GSK-3*β* (Ser9), P-GSK-3*β* (Tyr216), and *β*-catenin among the control group, sham-operated group, and negative control group. *α*-Syn relative protein expression in the PD model group was increased significantly compared with the control group (*P* < 0.01), and after 6 weeks of treatment with CFR in PD rats, *α*-syn expression was decreased significantly (*P* < 0.01). Similar tendency was also detected for the GSK-3*β* expression in groups. GSK-3*β* expression was significantly increased in the PD model group compared with the control group (*P* < 0.01), and with CFR administration in PD rats, the protein expression was decreased significantly (*P* < 0.05) compared to the PD model group. For P-GSK-3*β* (Ser9) and P-GSK-3*β* (Tyr216), opposite expression among the groups was observed. P-GSK-3*β* (Ser9) was significantly low-expressed in the PD model group compared with the control group (*P* < 0.01), and this decreased expression was increased in the PD + CFR treatment group (*P* < 0.01); compared with the control group, P-GSK-3*β* (Tyr216) expression was significantly increased in the PD model group (*P* < 0.01), and this expression level was inhibited in the PD + CFR treatment group (*P* < 0.05). The relative expression of *β*-catenin in the PD model group was significantly decreased compared with the control group (*P* < 0.01), and CFR administration into PD model rats significantly increased the expression of *β*-catenin (*P* < 0.01).

### 3.4. Effect of CFR on the SK-N-SH Cell Viability

**Figure 4:**
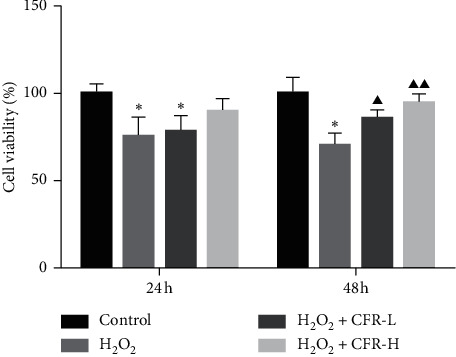
Effect of CFR on the SK-N-SH cell viability. Control group: transfected with empty control plasmids, H_2_O_2_ group: transfected with *α*-syn plasmids and 300 *μ*M H_2_O_2_, H_2_O_2_ + CFR-L group: transfected with *α*-syn plasmids, 300 *μ*M H_2_O_2_, and 5% CFR, and H_2_O_2_ + CFR-H group: transfected with *α*-syn plasmids, 300 *μ*M H_2_O_2_, and 10% CFR. Compared with the control group, ^*∗*^*P* < 0.05 and ^*∗∗*^*P* < 0.01; compared with the H_2_O_2_ group, ^*▲*^*P* < 0.05 and ^*▲▲*^*P* < 0.01.

After transfected with *α*-syn and incubated with H_2_O_2_, SK-N-SH cells were further treated with CFR for 24 h or 48 h, and CCK8 assay was used to assess the effect of CFR on cell viability. It could be observed that the cell viability in the H_2_O_2_ group decreased significantly compared with the control group in 24 h and 48 h incubation (*P* < 0.05, Figure 4). For 24 h of treatment with CFR in different dosages, the result showed that the cell viability in both H_2_O_2_ + CFR-L and H_2_O_2_ + CFR-H groups increased compared with the H_2_O_2_ group; however, no significant differences were observed. For 48 h of treatment with CFR in different dosages, the result showed that the cell viability in both H_2_O_2_ + CFR-L and H_2_O_2_ + CFR-H groups increased significantly compared with the H_2_O_2_ group (*P* < 0.05 and *P* < 0.01, respectively).

### 3.5. Effect of CFR on the Apoptosis of SK-N-SH Cells

**Figure 5:**
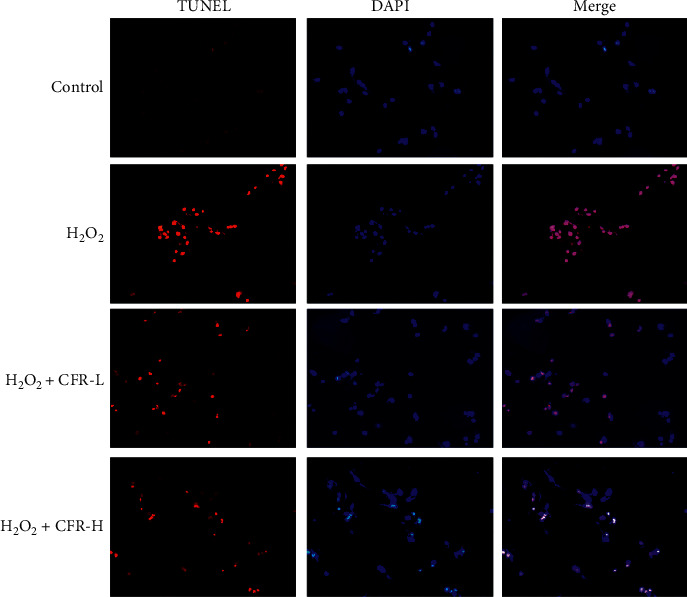
TUNEL assay for the effect of CFR on the apoptosis of the SK-N-SH cells (magnification, ×200). Control group: transfected with empty control plasmids, H_2_O_2_ group: transfected with *α*-syn plasmids and 300 *μ*M H_2_O_2_, H_2_O_2_ + CFR-L group: transfected with *α*-syn plasmids, 300 *μ*M H_2_O_2_, and 5% CFR, and H_2_O_2_ + CFR-H group: transfected with *α*-syn plasmids, 300 *μ*M H_2_O_2_, and 10% CFR.

TUNEL assay was performed to detect the effect of CFR on SK-N-SH cell apoptosis for 48 h treatment (Figure 5). Compared with the scarcely observed TUNEL-positive cells in the control group, TUNEL-positive cells in the H_2_O_2_ group were obviously increased, with high fluorescence intensity and amounts. After the CFR treatment, TUNEL-positive cells were decreased, and SK-N-SH cell apoptosis was inhibited in the H_2_O_2_ + CFR-L group and H_2_O_2_ + CFR-H group, especially in the H_2_O_2_ + CFR-H group compared with H_2_O_2_-treated cells.

### 3.6. Effect of CFR on the Expression of *α*-Syn in SK-N-SH Cells

**Figure 6:**
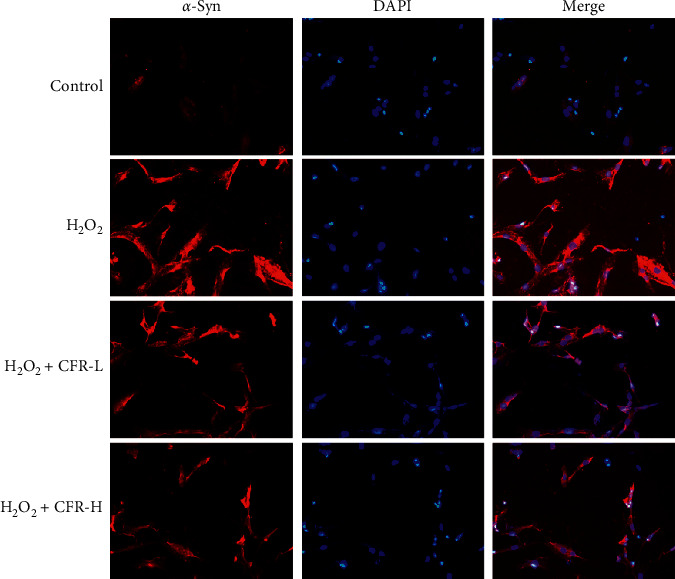
Expression of *α*-syn in SK-N-SH cells after the CFR treatment (magnification, ×200). Control group: transfected with empty control plasmids, H_2_O_2_ group: transfected with *α*-syn plasmids and 300 *μ*M H_2_O_2_, H_2_O_2_ + CFR-L group: transfected with *α*-syn plasmids, 300 *μ*M H_2_O_2_, and 5% CFR, and H_2_O_2_ + CFR-H group: transfected with *α*-syn plasmids, 300 *μ*M H_2_O_2_, and 10% CFR.

The effect of CFR on the expression of *α*-syn in SK-N-SH cells was also evaluated; the immunofluorescence results are shown in Figure 6. It could be found that *α*-syn was weakly expressed in the control group but was highly expressed in cytoplasm of the H_2_O_2_ group SK-N-SH cells, and the fluorescence intensity was significantly increased compared with the control group. In CFR treatment groups, low dose and high dose of CFR significantly decreased the fluorescence intensity of *α*-syn compared with the H_2_O_2_ group.

### 3.7. Effect of CFR on the Protein Expression of *α*-Syn, GSK-3*β*, P-GSK-3*β*, and *β*-Catenin in SK-N-SH Cells

**Figure 7:**
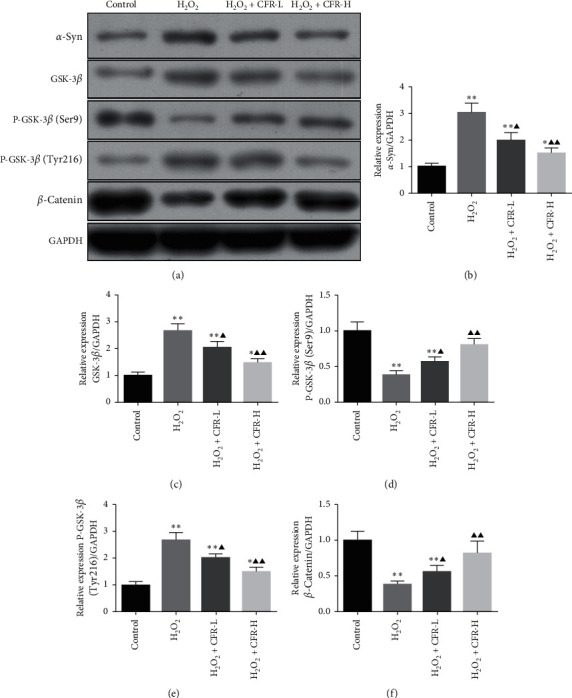
Effect of CFR on the protein expression of *α*-syn, GSK-3*β*, P-GSK-3*β* (Ser9), P-GSK-3*β* (Tyr216), and *β*-catenin in SK-N-SH cells. Control group: transfected with empty control plasmids, H_2_O_2_ group: transfected with *α*-syn plasmids and 300 *μ*M H_2_O_2_, H_2_O_2_ + CFR-L group: transfected with *α*-syn plasmids, 300 *μ*M H_2_O_2_, and 5% CFR, H_2_O_2_+CFR-H group: transfected with *α*-syn plasmids, 300 *μ*M H_2_O_2_, and 10% CFR. Protein expression was determined using western blot, and GAPDH was used as a control. Compared with the control group, ^*∗*^*P* < 0.05 and ^*∗∗*^*P* < 0.01; compared with the H_2_O_2_ group, ^*▲*^*P* < 0.05 and ^*▲▲*^*P* < 0.01.

As shown in Figure 7, the protein expression of *α*-syn, GSK-3*β*, and P-GSK-3*β* (Tyr216) in the H_2_O_2_ group was significantly increased compared with the control group (*P* < 0.01), while the protein expression of P-GSK-3*β* (Ser9) and *β*-catenin in the H_2_O_2_ group was significantly decreased compared with the control group (*P* < 0.01). Comparing these in the H_2_O_2_ group, the protein expression of *α*-syn, GSK-3*β*, and P-GSK-3*β* (Tyr216) in the H_2_O_2_ + CFR-L group and H_2_O_2_ + CFR-H group was significantly decreased (*P* < 0.05 and *P* < 0.01, respectively); and the protein expression of P-GSK-3*β* (Ser9) and *β*-catenin in the H_2_O_2_ + CFR-L group and H_2_O_2_ + CFR-H group was significantly increased (*P* < 0.05 and *P* < 0.01, respectively).

## 4. Discussion

As a common neurodegenerative disease, the diagnosis and management of PD remain challenging, and the choice of the drugs is still limited. Based on the critical role of DA in PD pathogenesis, neuroscientists revealed that PD could be reversed temporarily by pharmacologic interventions to restore dopaminergic neurotransmission [14]. As an intermediate in the pathway of dopamine synthesis, L-Dopa eventually becomes accepted as the preferred treatment for PD, but the adverse effects, dyskinesias, also occur in up to one-third of patients taking L-Dopa eventually [4, 15]. At the same time, more and more efforts have been made on exploring the new therapeutic strategies including gene therapy and stem cell transplantation therapy with the advances in the medical management, but the long-term curative effects and high cost also restrict the well clinical application of these therapies [16, 17].

The objective of the present study is to provide *in vivo* and *in vitro* evidence that CFR could be considered as a potential therapeutic agent in the model of PD. In this regard, the neuroprotective properties and possible mechanism of CFR were investigated in the AAV-*α*-syn-induced rat model of PD and SK-N-SH cells administrated with H_2_O_2_ and *α*-syn. In accordance with our previous research, the results showed that CFR significantly alleviated the neurotoxicity on PD rats and SK-N-SH cells. Besides, TUNEL assay and immunofluorescence staining assay also indicated that the abnormal accumulation of *α*-syn and increases of cell apoptosis in rats and SK-N-SH cells were also inhibited by CFR, and these regulation effects of CFR on PD might be correlated with the regulation of the GSK-3*β*/*β*-catenin signaling pathway.

CFR is composed by five traditional Chinese herbs: prepared *Rhizoma Rehmannia*, *Concha Margaritifera Usta*, fried *Salvia miltiorrhiza*, *Acorus gramineus Soland*, and green tea. Previous study shows that the active compound, salvianolic acid B, from *Salvia miltiorrhiza* can perform the prophylactic and therapeutic activities against neurodegenerative diseases including PD [18]. Pretreatment with salvianolic acid B in SH-SY5Y cells significantly reduces 6-hydroxydopamine- (6-OHDA-) induced generation of reactive oxygen species and prevents 6-OHDA-induced increases in intracellular calcium, which may be effective in treating neurodegenerative diseases [19]. And the tanshinone IIA from *Salvia miltiorrhiza* promotes the survival of DA neurons in the 1-methyl-4-phenyl-1,2,3,6-tetrahydropyridine (MPTP) mouse model of PD by the suppression of microglial activation and reduced expression of NADPH oxidase and iNOS [20]. Modified Yeoldahanso-tang including *Acorus gramineus Soland* was reported to have neuroprotective effects in *in vivo* and *in vitro* studies; it could inhibit both the loss of tyrosine hydroxylase- (TH-) positive neurons in the SNpc and the reduction of the optical density of tyrosine hydroxylase-immunoreactive (TH-IR) fibers in the striatum in a C57BL/6 mice model of PD [21]. Tea is one of the most consumed beverages worldwide, and multiple studies also prove that tea consumption has an inverse association with PD risk [22, 23]. Research studies focused on green tea show that the tea polyphenols have the profound neuroprotective and neuroregenerative effects; tea polyphenols do not just possess antioxidant or antichelating properties, but may directly interfere with aggregation of the *α*-syn protein and modulate intracellular signaling pathways, both in *in vitro* and animal models of PD [24].

In this experiment, overexpression of the disease-inducing protein *α*-syn was introduced for PD modeling. The progression of PD is mainly due to the degeneration of dopamine neurons in the SNpc. And as a main component of the Lewy bodies in dopamine neurons, abnormal accumulation of *α*-syn is proved to be responsible for the apoptosis of cells and the increasing possibility of neurodegeneration via the formation of the toxic forms like oligomer and polymer of *α*-syn [25, 26]. A previous study showed that storage, release, and reuptake of DA in the striatum are markedly impaired in *α*-syn-overexpressing DA neurons, suggesting that *α*-syn-induced axonal damage leads to synaptic DA function impairment [27]. Furthermore, different from the toxin-based models, like the injecting of 6-OHDA or MPTP in rodent and nonhuman primate animals, overexpression of *α*-syn has provided a novel PD model that recapitulates many features of the human disease. And comparison of the 6-OHDA and *α*-syn rat models in behavioral and histological characteristics revealed that DA neuron loss is prominent in both models, but the AAV-*α*-syn model replicates the human pathology more closely than other lesion models [28]. In this study, we used the *α*-syn-overexpressing model for *in vivo* and *in vitro* experiments. As shown in the results, the rotation numbers of PD rats were significantly increased comparing with those in the control group and sham-operated group rats; inhibition of cell viability and promotion of cell apoptosis were also observed in the *α*-syn-induced model group. Besides, the expression of *α*-syn was also elevated in the PD model rat and SK-N-SH cells, indicating the neurotoxicity role of *α*-syn in PD.

Accumulated evidence reveals that the disfunction of GSK-3*β* and its signaling cascades are directly related to the pathogenesis of neuroinflammation and neurodegenerative disorders [29]. GSK-3*β* contains two important sites, Ser9 and Tyr216, P-GSK-3*β* (Ser9) inhibits GSK-3*β* activity, and P-GSK-3*β* (Tyr216) promotes GSK-3*β* activity. Except for the important role in neural development including the regulation of neuronal apoptosis, studies found that GSK-3*β* has a mutual interaction with *α*-syn, which makes this kinase become an attractive therapeutic target for neurodegenerative disorders. It is reported that PD features such as *α*-syn aggregation and tau hyperphosphorylation induced by rotenone could be reversed by GSK-3*β*, and at the same time, *α*-syn regulates GSK-3*β* activity by decreasing Ser9 phosphorylation and elevating Tyr216 phosphorylation to mediate the neurotoxicity [30, 31]. Study also showed that Axin-2 shRNA- (negative regulator of Wnt/*β*-catenin signaling) mediated upregulation of Wnt/*β*-catenin signaling could enhance the net dopaminergic neurogenesis by improving behavioral functions and mitochondrial biogenesis in the SNpc, reducing apoptotic signaling, autophagy, and ROS generation, and regulating neural genes (Nurr-1, Pitx-3, Ngn-2, and NeuroD1) in PD rats [32]. In the present study, protein expression of GSK-3*β* and P-GSK-3*β* (Tyr216) was activated, while P-GSK-3*β* (Ser9) and *β*-catenin were inhibited in the PD model group with *α*-syn transferred, which was consistent with the previous study, and CFR administration significantly inhibited the overexpression of *α*-syn, GSK-3*β*, and P-GSK-3*β* (Tyr216) and also increased the protein expression of P-GSK-3*β* (Ser9) and *β*-catenin.

## 5. Conclusions

In conclusion, the present study was conducted to explore the protective effect of CFR against neurotoxicity and apoptosis induced by *α*-syn in *in vivo* and *in vitro* models of PD. As the *in vivo* study indicated, CFR attenuated the *α*-syn-induced PD rat behaviors with the rotation numbers decreased significantly and inhibited the cell apoptosis in the substantia nigra and striatum tissues of the PD rat brain; the overexpression of *α*-syn was inhibited, and the protein expression of GSK-3*β*, P-GSK-3*β* (Ser9), P-GSK-3*β* (Tyr216), and *β*-catenin in the GSK-3*β*/*β*-catenin signaling pathway was also regulated by CFR administration. Similar to the *in vivo* study, the *in vitro* study showed that CFR significantly improved the cell viability and inhibited SK-N-SH cell apoptosis; the inhibitory effect on *α*-syn and regulatory effect on GSK-3*β*, P-GSK-3*β* (Ser9), P-GSK-3*β* (Tyr216), and *β*-catenin of the GSK-3*β*/*β*-catenin signaling pathway were also observed in CFR-treated SK-N-SH cells. The present study suggested that CFR may be considered as a potential neuroprotective agent against PD, while further in-depth investigation is required.

## Data Availability

All data generated or analyzed during this study are included within this article.
